# Incidence of depressive symptoms and their associations with lifestyle and social support networks among community-dwelling older adults: a sex-stratified longitudinal study using the JAGES study

**DOI:** 10.1186/s13030-025-00342-y

**Published:** 2025-10-14

**Authors:** Mei Amaike, Ayuka Yokoyama, Yuko Tanaka, Momoka Yamazaki, Akemi Matsuzawa, Toshiyuki Ojima, Etsuko Tadaka

**Affiliations:** 1 Takikawa City, Hokkaido Japan; 2 Department of Community and Public Health Nursing, Faculty of Medicine/Graduate School of Health Sciences, Hokkaido University, Sapporo, Hokkaido Japan; 3 Department of Public Health Nursing, Faculty of Medicine/Graduate School of Health Sciences, Hokkaido University, Sapporo, Hokkaido Japan; 4 Department of Pediatric Nursing, Faculty of Medicine/Graduate School of Health Sciences, Hokkaido University, Sapporo, Hokkaido Japan; 5 Department of Community Health & Preventive Medicine, Hamamatsu University School of Medicine, Hamamatsu, Shizuoka Japan

**Keywords:** Depressive symptoms, Community-dwelling older adults, Lifestyle, Social support networks, Primary prevention

## Abstract

**Background:**

Primary prevention of depressive symptoms among independent older adults is a critical public health challenge. Lifestyle factors and social support networks are increasingly recognized as pivotal determinants of mental health in this population. However, few longitudinal studies have examined these relationships, and little attention has been directed to gender-based differences. We investigated the associations between lifestyle factors and social support networks and the depressive symptoms of community-dwelling independent older adults in a sex-stratified 3-year longitudinal study.

**Methods:**

This study used data from the Japan Gerontological Evaluation Study. Participants were functionally independent individuals aged 65 years and older. Our final analysis included data for 6,929 individuals collected between 2019 and 2022. A logistic regression analysis was conducted with depressive symptoms in 2022 as the dependent variable. Lifestyle factors and social support networks were treated as independent variables, with age, chronic diseases, and household income included as covariates. The effects of interactions between lifestyle factors and social support networks on depressive symptoms were also analyzed.

**Results:**

The prevalence of depressive symptoms was 10.7% in men and 11.6% in women in the 3-year follow-up. The incidence rate of newly developed depressive symptoms (per 1,000 person-years) was 35.6 for men and 38.8 for women. Sex-based differences were found in lifestyle and social support network factors associated with depressive symptoms. In men, walking for more than 30 min per day, insomnia, and emotional support were significantly associated with the risk for depressive symptoms. In women, walking for more than 30 min per day, insomnia, and instrumental support were significantly associated with the risk for depressive symptoms. No interaction effects between lifestyle factors and social support networks on depressive symptoms were observed in either men or women.

**Conclusion:**

These findings underscore the importance of preventing depressive symptoms among older adults, particularly through the lens of gender-specific differences in lifestyle factors and social support networks associated with depressive symptoms. This study highlights the potential for developing targeted and effective primary prevention strategies by identifying modifiable determinants of depressive symptoms.

## Background

Depressive symptoms are the most common mood disorder and have a significant impact on an individual’s socioeconomic status and quality of life. The World Health Organization (WHO) noted that approximately 14% of adults aged 60 years and above experienced mental health disorders in 2023, with 5.7% of these suffering from depressive symptoms [1]. In Japan, the highest proportion of individuals with mood disorders is in the group aged 65 years and older, which comprises 27.5% of the population. In addition, the number of older individuals with mood disorders increased from 270,000 in 2005 to 473,000 in 2020 (2020 Patient Survey) [2]. These reports indicated there is a growing number of older adults with depressive symptoms in Japan and in other developed countries with rapidly aging populations. The prevalence of depressive symptoms among individuals aged 65 years and above is rising [3], and both the prevalence and incidence of these symptoms increase with age [4]. Unlike depressive symptoms in younger adults, these symptoms in older adults are less likely to be associated with emotional symptoms. Instead, such symptoms often present as changes in cognitive function, physical symptoms, and loss of interest [5], and are frequently mistaken for signs of physical illnesses [6]. In primary care settings, sleep disorders and fatigue in older adults are often misdiagnosed as depressive symptoms, making it difficult to distinguish depressive symptoms from other age-related issues, such as reduced social interaction due to shrinking social networks [7].

Previous studies demonstrated that depressive symptoms in older adults contributed to health issues and other serious problems, including coronary artery disease and stroke [8], decreased activities of daily living (ADL) and motor function [9], social isolation [10], and cognitive decline [11]. Notably, 71%–97% of suicides among older adults are associated with mental disorders, with mood disorders, particularly depressive symptoms, being the most common [12]. These findings underscore the major impact of depressive symptoms on older adults’ overall health and prognosis and highlight the importance of early detection and prevention.

Most available research on factors contributing to depressive symptoms among community-dwelling older adults focused on sociodemographic characteristics. The relationship between lifestyle habits and depressive symptoms has also been explored. For example, high-protein intake, which provides essential amino acids necessary for neurotransmitter production, has been suggested to be inversely correlated with depressive symptoms [13]. Physical activity has also been investigated, with moderate to high-intensity exercise once or twice a week shown to reduce depressive symptoms [14–16]. Furthermore, insomnia has been strongly linked to depressive symptoms [17].Social support networks have also been found to play a role in preventing depressive symptoms. A systematic review of studies involving older adults in Asian communities found that emotional and instrumental support significantly reduced depressive symptoms [18]. Communication and mutual support with family members, neighbors, and friends have been shown to contribute to mental health [19]. Strong social support networks have a protective effect against depressive symptoms, meaning these networks are important for the mental well-being of community-dwelling older adults [14]. Furthermore, although older adults with fewer opportunities for instrumental support are at high risk for depressive symptoms, expecting support from others (not just a spouse) has been found to effectively reduce depressive symptoms [20].

Unfortunately, past research has been insufficient in identifying predictors to support primary prevention of depressive symptoms among older adults. There are four main reasons for this gap. First, studies focused on modifiable lifestyle factors as intervention targets are limited. Second, despite the fact that physical activity, nutrition, and sleep are interrelated, no studies comprehensively examined these factors simultaneously. Third, although social support networks act as a protective factor against depressive symptoms [21, 22], there is a lack of research exploring their interaction with lifestyle habits. Finally, although the risk for depressive symptoms differs by sex [23–25], the ways in which gender differences in lifestyle habits and social support network systems are related to depressive symptoms have not been adequately clarified [26]. Given these gaps, it is necessary to investigate the relationship between depressive symptoms and both lifestyle habits and social support networks by sex. Such research could provide valuable insights for developing gender-specific public health strategies aimed at the primary prevention of depressive symptoms among older adults.

This study explored the relationship between depressive symptoms and lifestyle habits and social support networks among community-dwelling older adults, with a focus on gender. The results are intended to contribute to the development of guidelines focused on lifestyle habits and social support networks for the primary prevention of depressive symptoms among community-dwelling older adults.

## Methods

### Study design

This was a population-based longitudinal study conducted in Japan as part of the Japan Gerontological Evaluation Study (JAGES). JAGES data were accessed for the purpose of this study on July 1, 2024.

### Participants

Participants in this study were individuals enrolled in the JAGES, which is a large-scale cohort study [27]. The JAGES included community-dwelling individuals aged 65 years and older living in 76 municipalities across Japan, none of whom were certified as requiring long-term care. An anonymous self-administered questionnaire was mailed to approximately 230,000 participants between 2019 and 2022 [28]. Survey participants were selected on a city, town, or village basis. A list of people aged 65 years or over was created based on either the list of primary long-term care insurance beneficiaries or the basic resident register, whichever was easier for the local government to use. This list was used as a sampling frame. Those in need of long-term care were excluded based on information from long-term care insurance nursing care certification data. Questionnaires were then distributed to all participants in cities, towns, or villages with a population less than 5,000 (as a general rule), and to people selected randomly in cities, towns, or villages with a population of 5,000 or more. Participants typically completed the questionnaire independently. The 2019 survey targeted 66 municipalities and was conducted from November 2019 to January 2020. The 2022 survey targeted 76 municipalities and was conducted from November 2022 to December 2022. In principle, the questionnaire comprised basic items distributed to all eligible individuals, along with different versions randomly assigned to selected participants. For this study, the version containing items related to sleep was selected from the eight available versions.

### Exclusion criteria

The following were excluded from this study. a) Individuals who did not respond to the 2022 follow-up survey. b) Individuals who showed depressive tendencies (Geriatric Depressive Symptoms Scale [GDS]−15 score ≥ 5) or had missing data in the 2019 baseline survey (because the present study focused on the primary prevention of depressive symptoms). c) Individuals whose age or gender in the 2019 baseline survey did not match the 2022 follow-up survey. d) Those who required assistance with ADL or long-term care at the time of the 2019 baseline survey. e) Individuals with missing GDS-15 data in the 2022 follow-up survey.

### Research framework

**Fig. 1:**
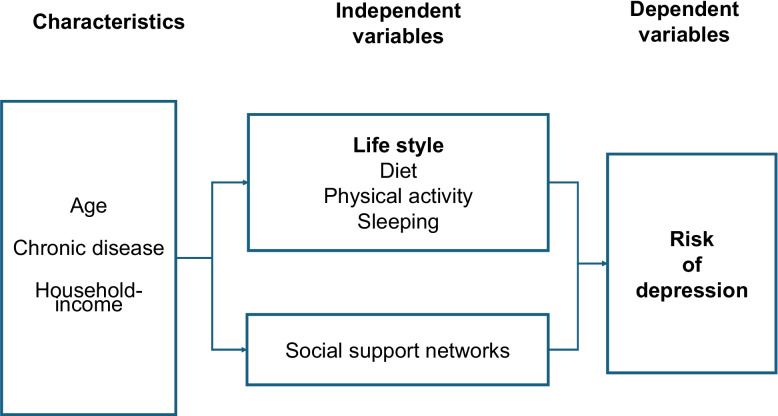
Research framework

This study included basic attributes such as age, family composition, chronic conditions, household income, and educational background. Lifestyle habits and social support networks were treated as independent variables in the analysis, with depressive symptoms as the dependent variable. In addition to the direct effects of lifestyle habits and social support networks on the onset of depressive symptoms, the presence of social support networks may have an additional effect in reducing the onset of depressive symptoms through synergistic interactions with lifestyle factors. Therefore, we hypothesized that the impact of lifestyle factors on the onset of depressive symptoms may vary depending on the presence of social support networks. A further analysis was conducted to explore the interaction between lifestyle factors and social support networks and its effect on the onset of depressive symptoms Fig. 1.

## Dependent variable

### Depressive symptoms at follow-up (2022)

Depressive symptoms were assessed using the GDS-15 in both the baseline and follow-up surveys. The GDS-15 is a widely used tool for screening depressive symptoms in older adult populations. In a previous study, the pooled sensitivity and specificity of the GDS-15 were 0.89 and 0.77, respectively [29]. GDS-15 scores range from 0 to 15, with scores of 0–4 indicating no depressive symptoms and scores of ≥ 5 indicating depressive symptoms [30–32]. In this study, scores of 0–4 were classified as “no depressive symptoms” and scores of ≥ 5 as “depressive symptoms.”

## Independent variables

### Lifestyle factors at baseline (2019)

Based on prior research, three lifestyle variables thought to be associated with the risk for depressive symptoms, each with individual and interaction effects, were included in this study. These variables were diet, which contributes to neurotransmitters and is known to increase depressive symptoms risk because of nutritional deficiencies [33–35], physical activity [36–38], and sleep [39, 40].

### Diet (Daily Protein Intake)

Japan’s Ministry of Health, Labour and Welfare has promoted protein intake to extend the healthy lifespan of older adults [41]. In addition to the social background in which the importance of protein intake for older adults is increasing, protein plays a major role in the mechanism underlying depression (i.e., insufficient protein intake results in insufficient synthesis of neurotransmitters from amino acids, leading to depression) [42]. First, the frequency of meat and fish consumption has been utilized in the National Health and Nutrition Survey in Japan [43], and the binary (two-category) response format has long been widely adopted in large-scale epidemiological studies [44], thereby facilitating comparability across studies. Furthermore, previous research has demonstrated that meat and fish intake is strongly associated with total protein intake, indicating that frequency of meat and fish consumption serves as a reasonable proxy for protein intake [45, 46]. In addition, the Japan Gerontological Evaluation Study (JAGES) was designed as a self-administered survey targeting large populations of community-dwelling older adults across Japan. Within this context, a simplified frequency-based classification was considered the most practical approach to ensure standardized measurement nationwide, minimize respondent burden, and achieve high response rates.

Therefore, this study investigated the frequency of protein intake using the following question. “In the past month, how frequently did you eat meat or fish?” The response options were: “More than twice a day,” “Once a day,” “4–6 times a week,” “2–3 times a week,” “Once a week,” “Less than once a week,” and “Never.” Participants who answered “More than twice a day” or “Once a day” were classified as the high-frequency group, with all other responses classified as the low-frequency group [47, 48].

### Physical activity (Daily Walking Time)

Based on previous studies and guidelines from the WHO [49]and the Ministry of Health, Labour and Welfare [50], average daily walking time was assessed with the question: “On average, how much do you walk each day?” The response options were: “Less than 30 min,” “30–59 min,” “60–89 min,” and “90 min or more.” Participants who answered “Less than 30 min” were classified as the low-frequency group, and all others were classified as the high-frequency group [51, 52].

### Sleep

The presence of sleep disturbance was assessed using the Japanese version of the Athens Insomnia Scale. Scores of 0–3 were classified as “no insomnia,” and scores of ≥ 4 were classified as “insomnia present” [53].

### Social support networks at baseline (2019)

Social support networks were included in this study because prior studies showed that older adults with strong social support networks had a lower prevalence of depressive symptoms than those without such networks and because social support networks played a crucial role in the primary prevention of depressive symptoms [19, 22, 54]. Therefore, we hypothesized that social support networks help prevent and reduce depressive symptoms.

## Social support networks

In this study, individuals who responded "yes" to either providing or receiving support were defined as having Social Support Networks. More specifically, participants who indicated that they either provided or received support from "friends" or "neighbors" were categorized as possessing such networks. The focus of this study was not on the type of support received by community-dwelling, independent older adults, but rather on the presence or absence of support. Both policy and academic considerations guided this approach. For example, the revised edition of the Depression Prevention and Support Manual issued by the Ministry of Health, Labour, and Welfare highlights the importance of mutual aid among neighbors in preventing depression in older adults. Furthermore, expectations and obligations associated with familial roles are typically more clearly defined compared to the social roles embedded in non-familial relationships, such as those with friends or colleagues. Consequently, within familial contexts, the act of providing support may have a more detrimental effect on health than receiving support [55], while providing emotional or instrumental support to individuals outside the family has been linked to reductions in depressive symptoms [56]***.***We investigated the presence or absence of emotional support and instrumental support as part of social support. We used two questions to assess emotional support: “Do you have someone who listens to your concerns and complaints?” and “Do you listen to someone else’s concerns or complaints?” The response options for these questions were “Spouse,” “Children living together,” “Children or relatives living separately,” “Siblings, relatives, parents, grandchildren,” “Neighbors,” “Friends,” “Other,” and “No one like that.” Participants who answered “Neighbors” or “Friends” to either of these questions were classified as receiving emotional support, with all other answers classified as not receiving support [57–59]. Two questions were used to assess instrumental support: “Do you have someone who looks after you when you are sick and confined to bed for a few days?” and “Do you look after someone else when they are sick and confined to bed for a few days?” The response options were the same as for the emotional support questions. Participants who answered “Neighbors” or “Friends” were classified as receiving instrumental support, and all other answers as not receiving support [57–59]. We excluded family support, because giving and receiving support from family members may be influenced by whether or an individual lives with those family members. In addition, mutual aid is increasingly promoted in Japanese society, whereby neighbors and friends help each other.

### Covariates

Based on previous studies [60–63], the covariates assessed were: age (65–74 years, ≥ 75 years), ever having had any illness or current chronic disease (none, yes), household income (< 2.0 million yen/year, 2.0–3.99 million yen/year, ≥ 4.0 million yen/year). These variables were all measured in the 2019 baseline survey.

### Statistical analyses

First, descriptive statistics were used to characterize study participants. They were then stratified by sex, and chi-square tests were performed to examine the relationships between the presence of depressive symptoms and individual lifestyle and social support network characteristics.

Next, logistic regression analyses were conducted separately for men and women to analyze the influence of baseline lifestyle and social support networks on the development of depressive symptoms. Variables that were significantly associated with depressive symptoms were included in the analysis. Univariate analyses, including covariates, were conducted with consideration of multicollinearity. Odds ratios (ORs) and 95% confidence intervals (95% CIs) for the risk for depressive symptoms were calculated. The dependent variable was the presence of new depressive symptoms at the 2022 follow-up (0 = no depressive symptoms, 1 = depressive symptoms). The independent variables were lifestyle factors (eating habits, physical activity, sleep patterns) and social support networks (emotional and instrumental social support networks). Baseline demographic attributes were included as covariates.

Furthermore, logistic regression models were employed to examine the effect of any interactions between lifestyle habits and social support networks on depressive symptoms, with interaction terms included separately for men and women. Specifically, variables identified in the univariate logistic regression analysis as significantly associated with depressive symptoms were analyzed (i.e., the interactions between walking and emotional support for men and walking and instrumental support for women). In this study, an available case analysis was conducted in which no imputation for missing data was performed, and only cases with complete data on all explanatory variables were included in the analysis. Missing data were excluded because single imputation methods do not sufficiently account for the uncertainty inherent in missing values and may introduce bias into the estimates. Because of the potential impact of the COVID-19 pandemic between 2019 and 2022, a supplementary cross-sectional analysis using baseline (2019) data was performed to verify the validity of the longitudinal findings. All statistical analyses were performed using SPSS (version 26.0, IBM Corp., Armonk, NY, USA), with the significance level set at 5%.

## Results

**Fig. 2:**
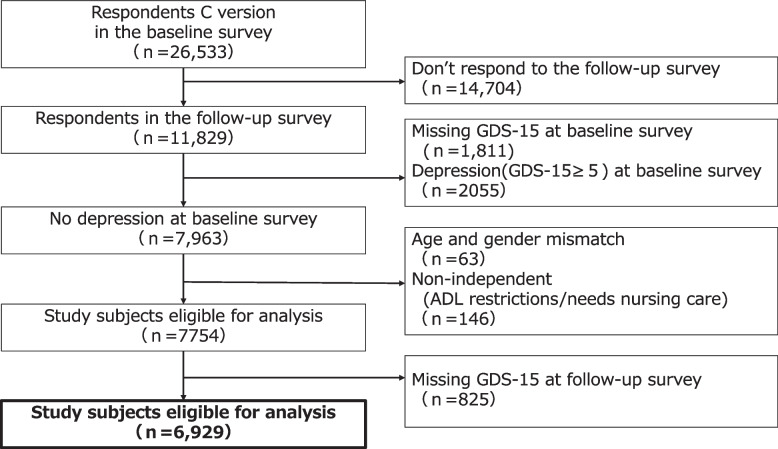
Flowchart of study participants

In total, 11,829 participants completed both the 2019 baseline survey and the follow-up survey in 2022. After excluding individuals based on the exclusion criteria, the final sample for analysis in this study comprised 6,929 participants Fig. 2.

The overall prevalence of depressive symptoms in the 3-year follow-up of this study was 11.2% (10.7% in men and 11.6% in women). Of the 3,544 participating men, 379 developed depressive symptoms. The incidence of depressive symptoms among independent older men living in the community was 35.6 cases per 1,000 person-years. Of the 3,385 participating women, 394 developed depressive symptoms. The incidence of depressive symptoms among independent older women living in the community was 38.8 cases per 1,000 person-years.

Table 1 presents the characteristics of the participants by sex. We observed significant differences in sociodemographic characteristics between men and women in terms of household income, daily protein intake, insomnia, emotional support networks, and instrumental support networks.

**Table 1:** The characteristics of the participants by sex (*n* = 6,929)

Characteristics	Men(*n* = 3544)		Women(*n* = 3385)		*P* value
	N	%	N	%	
Depressive symptoms	(*n* = 3544)		(*n* = 3385)		0.222
No	3165	89.3	2991	88.4	
Yes	379	10.7	394	11.6	
Age yrs	(*n* = 3544)		(*n* = 3385)		0.399
65–74	1619	45.7	1581	46.7	
75 <	1925	54.3	1804	53.3	
Chronic disease	(*n* = 3413)		(*n* = 3183)		0.937
Absence	1109	32.5	1038	32.6	
Presence	2304	67.5	2145	67.4	
Household income JPY10,000	(*n* = 3280)		(*n* = 2924)		< 0.001**
< 200	439	13.4	673	23.0	
200 ~ 400	1468	44.8	1171	40.0	
400 <	1373	41.9	1080	36.9	
Daily Protein Intake	(*n* = 3468)		(*n* = 3324)		< 0.001**
no	1707	49.2	1245	37.5	
yes	1761	50.8	2079	62.5	
Daily Walking time	(*n* = 3544)		(*n* = 3385)		0.305
< 30 min	802	22.6	802	23.7	
> 30 min	2742	77.4	2583	76.3	
Insomnia	(*n* = 3348)		(*n* = 3100)		< 0.001**
no	2296	68.6	1972	63.6	
yes	1052	31.4	1128	36.4	
Emotional support	(*n* = 3482)		(*n* = 3338)		< 0.001**
no	1846	53.0	824	24.7	
yes	1636	47.0	2514	75.3	
Instrumental support	(*n* = 3458)		(*n* = 3285)		< 0.001**
no	3189	92.2	2679	81.6	
yes	269	7.8	606	18.4	

Data from participants who had missing values were excluded from the analysis

^*^p < 0.05

**p < 0.001

Table 2 shows the factors that influenced the depressive symptoms of men. Men aged 65–74 years (OR 1.554, 95% CI: 1.224–1.974) had significantly higher odds of having depressive symptoms compared with those aged ≥ 75 years. Men with chronic diseases had higher odds of having depressive symptoms compared with those without chronic diseases (OR 1.554, 95% CI: 1.193–2.026). Those in the household income brackets 2.0–4.0 million yen (OR 0.697, 95% CI: 0.511–0.952) and over 4.0 million yen (OR 0.51, 95% CI: 0.367–0.710) had significantly lower odds of having depressive symptoms compared with those with a household income under 2.0 million yen.

**Table 2:** Effects of lifestyle and social support networks on the risk of depressive symptoms in men (*n* = 3,544)

	OR	95%CI	*p*-value
Characteristics			
Age			
65–74	ref		
75-	1.55	1.22–1.97	< 0.001**
Chronic disease			
absence	ref		
presence	1.55	1.19–2.03	0.001*
Household income JPY 10,000			
< 200	ref		
200–400	0.70	0.51–0.95	0.023*
400 <	0.51	0.37–0.71	< 0.001**
Lifestyle Factors ^a)^			
Daily Protein Intake			
no	ref		
yes	0.82	0.65–1.04	0.096
> Daily 30 min walking			
no	ref		
yes	0.69	0.54–0.89	0.004*
Insomnia			
no	ref		
yes	1.82	1.43–2.31	< 0.001**
Social support network^a)^			
Emotional support			
no	ref		
yes	0.64	0.51–0.81	< 0.001**
Instrumental support			
no	ref		
yes	0.65	0.40–1.05	0.077
Lifestyle Social Support Networks Interaction ^a)^			
Daily 30 min walk * emotional support			
no	ref		
yes	0.83	0.49–1.40	0.478

Data from participants who had missing values were excluded from the analysis

^*^p < 0.05

**p < 0.001

^a)^ Adjusted for characteristics (Age, Chronic disease, Household income)

The analysis of lifestyle factors showed that men who walked for more than 30 min per day had significantly lower odds of having depressive symptoms compared with those who walked less than 30 min/day (OR 0.692, 95% CI: 0.537–0.892). Finally, men with insomnia had significantly higher odds of having depressive symptoms compared with those without insomnia (OR 1.821, 95% CI: 1.434–2.313).

Investigation of social support networks showed that men with emotional support networks had significantly lower odds of having depressive symptoms compared with those without emotional support networks (OR 0.641, 95% CI: 0.506–0.813). However, no significant association was found between depressive symptoms and the interaction between 30 min of walking/day and emotional support.

To verify the validity of the longitudinal results, an additional cross-sectional analysis was conducted using the 2019 baseline data. These findings were consistent with the longitudinal analysis from 2019 to 2022. Specifically, among 4,720 men, chronic diseases, household income, daily protein intake, daily walking time, insomnia, emotional support, and instrumental support were significantly associated with depressive symptoms. However, no significant association was found between depressive symptoms and the interaction between 30 min of walking/day and emotional support.

Table 3 shows the factors that influenced the depressive symptoms of women. Examination of sociodemographic characteristics showed that women with chronic diseases had higher odds of having depressive symptoms compared with those without chronic diseases (OR 1.383, 95% CI: 1.055–1.812). Those with household incomes of 2.0–4.0 million yen (OR 0.623, 95% CI: 0.471–0.825) and over 4.0 million yen (OR 0.401, 95% CI: 0.292–0.552) had significantly lower odds of having depressive symptoms compared with those with a household income under 2.0 million yen.

**Table 3:** Effects of lifestyle and social support networks on the risk of depressive symptoms in women (*n* = 3,385)

	OR	95%CI	*p* value
Characteristics			
Age			
65–74	ref		
75-	1.01	0.79–1.30	0.926
Chronic disease			
absence	ref		
presence	1.38	1.06–1.81	0.001*
Household income JPY 10,000			
< 200	ref		
200–400	0.62	0.47–0.83	0.001*
400 <	0.40	0.29–0.55	< 0.001**
Lifestyle Factors ^a)^			
Daily Protein Intake			
no	ref		
yes	0.88	0.68–1.13	0.30
> Daily 30 min walking			
no	ref		
yes	0.66	0.51–0.85	0.002*
Insomnia			
no	ref		
yes	1.41	1.10–1.82	0.008*
Social support networks ^a)^			
Emotional support			
no	ref		
yes	0.80	0.62–1.05	0.110
Instrumental support			
no	ref		
yes	0.46	0.31–0.68	< 0.001**
Lifestyle social support network interaction ^a)^			
Daily 30 min walk * Instrumental support			
no	ref		
yes	1.35	0.56–3.27	0.505

Data from participants who had missing values were excluded from the analysis

^*^p < 0.05

**p < 0.001

^a)^ Adjusted for characteristics (Age, Chronic disease, Household income)

Women who walked for more than 30 min per day had significantly lower odds of having depressive symptoms compared with those who walked for less than 30 min/day (OR 0.658, 95% CI: 1.094–1.821). Finally, women with insomnia had significantly higher odds of having depressive symptoms compared with those without insomnia (OR 1.411, 95% CI: 1.094–1.821).

The analysis of social support networks showed that women with instrumental support had significantly lower odds of having depressive symptoms compared with those without this support (OR 0.46, 95% CI: 0.312–0.678). However, no significant association was found between depressive symptoms and the interaction between 30 min of walking per day and instrumental support.

To verify the validity of the longitudinal results, an additional cross-sectional analysis was conducted using the 2019 baseline data. These findings were consistent with the longitudinal analysis from 2019 to 2022. Specifically, among 4,577 women, age, chronic disease, household income, daily protein intake, daily walking time, insomnia, emotional support, and instrumental support were significantly associated with depressive symptoms. However, no significant association was found between depressive symptoms and the interaction between 30 min of walking per day and instrumental support.

## Discussion

This study offers original insights based on the large-scale JAGES dataset, which explored the 3-year risk for depressive symptoms among community-dwelling older adults in Japan. By examining lifestyle habits and social support networks, we identified key factors that influenced the onset of depressive symptoms in this demographic.

### Depressive symptoms among community-dwelling independent older adults by sex

This study included 3,544 men and 3,385 women, of which 379 men and 394 women developed depressive symptoms over 3 years. This yielded an overall prevalence of depressive symptoms of 11.2% (10.7% in men and 11.6% in women). In Japan, large-scale cohort studies found prevalence rates of 10.4% [64] and 10.7% [65], which were lower than those reported in international studies (e.g., 31.7% [66], 35.1% [67], and 28.4% [68]) but consistent with the findings of this study. The differences in prevalence rates may stem from the inclusion of institutionalized or hospitalized patients in international studies. Furthermore, Japan possesses a comprehensive healthcare and welfare infrastructure accompanied by government-led initiatives targeting mental health. These factors may contribute to the relatively low prevalence of depressive symptoms in comparison to other countries.

The incidence of depressive symptoms (per person-years) was 36 per 1,000 person-years for men and 39 per 1,000 person-years for women. Other longitudinal studies conducted in Japan have reported incidence rates ranging from 33.9 to 35.4 cases per 1,000 person-years [64, 69], indicating higher prevalence compared to international data (e.g., 19.9 cases [70], and 18.8 cases [71] per 1,000 person-years). However, this study found the incidence in Japan was higher than that observed in both developed and emerging countries. Other longitudinal studies conducted in Japan reported similar incidence rates of 33.9–35.4 per 1,000 person-years [64, 69]. This higher incidence may be attributed to Japan’s large proportion of older adults as well as to lifestyle factors such as the prevalence of sleep disorders, which is notably higher in Japan (34%) [72] compared with that in the United States (18.3%) [73].

Given Japan’s aging population, the number of older adults developing depressive symptoms is expected to rise. In 2023, Japan’s aged rate was 29.1%, with an estimated 570,000 men and 800,000 women projected to develop depressive symptoms annually. This underscores the critical need to prioritize preventive measures for this high-risk population.

### Effects of lifestyle habits and social support networks on depressive symptoms by sex

This study revealed gender-specific differences in the risk factors for depressive symptoms related to lifestyle habits and social support networks. For both men and women, walking for more than 30 min/day was significantly associated with a reduced risk for depressive symptoms, whereas insomnia was linked to an increased risk. Physical activity, such as walking, is known to elevate serotonin levels and promote neurogenesis, which helps mitigate the risk for depression [74]. Conversely, insomnia increases anxiety and impairs brain function, thereby heightening the risk for depression [75]. Therefore, promoting walking and preventing insomnia are essential strategies for primary prevention of depressive symptoms in community-dwelling, independent older adults.

This study further revealed that emotional support was associated with a reduced risk for depressive symptoms in men, whereas instrumental support had a more significant impact for women. Previous research indicated that men were more likely to rely on instrumental support for practical tasks, whereas women tended to seek emotional support, particularly from sources outside the family [76, 77]. In Japan, traditional gender roles may influence these patterns. Older men more likely to provide instrumental support, with women being more inclined to both offer and receive emotional support. In this study, older men who received emotional support from outside the family exhibited a lower risk for depressive symptoms, whereas emotional support had a less pronounced effect on women because of its broader availability within the family. However, instrumental support proved more beneficial for women in preventing depressive symptoms in this study. This may be attributable to women’s greater reliance on external support for tasks such as driving, as they are more likely to seek assistance than men [78]. Women who received instrumental support at least once a week had a lower risk for developing depressive symptoms compared with those who did not receive such support [79, 80].

This study found no significant interaction effects between lifestyle habits and social support networks in terms of preventing depressive symptoms. Previous studies have demonstrated the moderating role of social support in mitigating depression resulting from functional impairments and stress among older adults [81, 82]. However, interaction effects involving other factors have not been empirically established. This may indicate that social support is particularly effective in counteracting biologically driven causes of depression, which could explain the absence of interaction effects with behavioral factors such as lifestyle habits in the present study. Moreover, prior research has suggested that the lack of statistically significant interaction effects may be attributable to limitations in measuring social support with a single item and to insufficient sample size [83]. Similarly, in this study, social support networks were assessed solely based on the presence or absence of support from neighbors and friends. Therefore, future research should adopt broader metrics, such as the Social Capital (SC) indicators developed by JAGES, and employ larger sample sizes to facilitate more robust interaction analyses. However, both factors independently influenced the onset of depressive symptoms. For example, engaging in physical activity or receiving social support can reduce the risk for depression even among those with physical limitations, which suggests that personalized prevention strategies may be effective.

This study has three main limitations. First, the data were self-reported, which may have led to either under- or overestimation of certain factors. Second, the study period overlapped the COVID-19 pandemic, which might have influenced the results. As part of a sensitivity analysis, an additional cross-sectional analysis was conducted using the 2019 baseline dataset, which included 4,720 men and 4,577 women from this study. The results were consistent with those obtained from the main dataset of the study (2019–2022), thereby providing some validation of the study's findings. However, it remains plausible that individuals most severely affected by the COVID-19 pandemic may have dropped out by 2022, potentially introducing survivor bias.

The pandemic could have resulted in either under- or overestimation of the risk for depressive symptoms, depending on whether participants dropped out or reported heightened symptoms due to pandemic-related stress. Finally, potential limitations and associations with unmeasured factors should be considered. One possible explanation for the significantly lower household income among female participants compared to their male counterparts may be that "household income" refers to joint income shared with a spouse. Marital status is a well-established determinant of depression [84]; however, it was not assessed in the present study. Thus, marital status may have acted as an underlying factor contributing to the observed income differences. Additionally, regional factors such as sunlight exposure [85] and disparities between urban and rural areas [86] have been shown to influence the onset of depressive symptoms. These factors warrant further investigation in future research.

## Conclusion

This study employed a large-scale dataset to examine the 3-year risk for depressive symptoms among independent older adults and explore the roles of lifestyle habits and social support networks. The findings highlight the importance of preventive measures for depressive symptoms in older adults, particularly through gender-specific interventions targeting modifiable risk factors. Further research should focus on subgroup analyses based on household types and residential areas, as well as deeper investigation of secondary and tertiary prevention strategies.

## Data Availability

The data that support the findings of this study are available from the Japan Agency for Gerontological Evaluation Study (URL: https://www.jages.net/). However, restrictions apply to the availability of these data, which were used under license for the current study, and so are not publicly available. Data are however available from the authors upon reasonable request and with the permission of the Japan Agency for Gerontological Evaluation Study (URL: https://www.jages.net/). All inquiries regarding the data are to be addressed to the data management committee via e-mail: dataadmin.ml@jages.net.
